# Development and Characterization of Mycelium-Based Composite Using Agro-Industrial Waste and *Ganoderma lucidum* as Insulating Material

**DOI:** 10.3390/jof11060460

**Published:** 2025-06-17

**Authors:** Gustavo Jiménez-Obando, Juan Sebastian Arcila, Ricardo Augusto Tolosa-Correa, Yenny Leandra Valencia-Cardona, Sandra Montoya

**Affiliations:** 1 Escuela de Arquitectura y Urbanismo, Universidad Nacional de Colombia, Calle 65 # 23-29, Manizales 170004, Colombia; gjimenezob@unal.edu.co (G.J.-O.); ratolosac@unal.edu.co (R.A.T.-C.); 2 GESA Grupo de Estudios en Sostenibilidad Ambiental, Universidad Nacional de Colombia, Carrera 45 # 26-85, Bogotá 11001, Colombia; 3 Center for Technological Development—Bioprocess and Agro-Industry Plant, Department of Engineering, Universidad de Caldas, Calle 65 # 26-10, Manizales 170004, Colombia; juan.arcila@ucaldas.edu.co (J.S.A.); leandra.valenciacardona@udecaldas.edu.co (Y.L.V.-C.); 4 Grupo de Procesos Químicos, Catalíticos y Biotecnológicos, Universidad Nacional de Colombia, Campus La Nubia, Bloque T, Km 7 vía al Aeropuerto, Manizales 170003, Colombia

**Keywords:** mycelium-based composite, *Ganoderma lucidum*, thermal insulation, *Montanoa quadrangularis* Shultz Bip

## Abstract

Mycelium-based composites (MBCs) have emerged as eco-friendly alternatives, utilizing fungal mycelium as a natural binder for agro-industrial residues. This study focuses on developing an MBC based on abundant waste in Colombia, pith Arboloco (A) (*Montanoa quadrangularis*), a plant endemic to the Colombian–Venezuelan Andes with outstanding insulating properties, and natural fiber of Kikuyu grass (G) (*Cenchrus clandestinus*), utilizing *Ganoderma lucidum* as an agent to form a mycelium network in the MBC. Three formulations, T (100% A), F1 (70% A/30% G), and F2 (30% A/70% G), were evaluated under two different Arboloco particle size ranges (1.0 to 5.6 mm) for their physical, mechanical, and thermal properties. The Arboloco particle sizes did not show significant differences in the MBC properties. An increase in Kikuyu grass proportion (F2) demonstrated superior density (60.4 ± 4.5 kg/m^3^), lower water absorption (56.6 ± 18.4%), and better compressive strength (0.1686 MPa at 50% deformation). Both mixing formulations (F1–F2) achieved promising average thermal conductivity and specific heat capacity values of 0.047 ± 0.002 W m^−1^ K^−1^ and 1714 ± 105 J kg^−1^ K^−1^, comparable to commercial insulation materials. However, significant shrinkage (up to 53.6%) and high water absorption limit their scalability for broader applications. These findings enhance the understanding of MBC’s potential for non-structural building materials made of regional lignocellulosic waste, promoting a circular economy in waste management for developing countries.

## 1. Introduction

The construction industry represents one of the sectors with the highest energy consumption globally. It utilizes approximately 40% of the energy generated annually during the various stages of its life cycle, including manufacturing, on-site construction, and final disposal of materials. The demand for construction materials has grown 23 times, and it is estimated that between 2017 and 2026, the number of square meters built in buildings worldwide will increase from 163 to 184 billion [1]. This trend presents a challenging outlook from both economic and environmental perspectives, where eco-design and engineering play a crucial role in developing sustainable building alternatives that minimize the environmental impact associated with greenhouse gas emissions, energy consumption, water pollution, and waste inherent in the production and transportation of building materials [2]. The evolution of sustainable building materials traces a path from traditional practices to cutting-edge innovation. The pioneering adoption of renewable resources, such as bamboo [3] and wood [4,5], has been characterized by their low environmental impact and structural adaptability. This foundation shifted toward circular economy principles, incorporating recycled materials such as crushed concrete, plastic, and glass as aggregates in concrete [6,7,8]; the development of novel materials based on the inclusion of steel fibers as a reinforced agent to form High-Toughness Recycled Aggregate Concrete (HTRAC) [9]; or the use of recycled brick powder-based geopolymer brick powder as a precursor to prepare alkali-activated based geopolymers (AABGs) [10], which led to the reduction of waste and conserving resources without substantially affecting the strength, deformability, and toughness properties of concrete.

Concomitantly with global warming changes and its sharp and long outdoor temperature fluctuation, the external envelope of a building has become a crucial aspect in eco-design projects due to the necessity of controlling the surrounding microclimate, which is directly associated with the thermal comfort of inhabitants and energy loss, which decreases energy consumption due to heating and cooling demands [11]. Mycelium-based composites (MBCs) are emerging as promising insulating materials due to their sustainable and effective thermal properties. White-rot fungi (WRT), a primary fungal species in MBC production, preferentially utilize agro-industrial residues due to their lignocellulosic matrix composition [12]. The activation of the enzymatic machinery of white-rot fungi through the production of laccase, manganese peroxidases (MnP), and lignin peroxidases (LiP) breaks down lignin while preserving cellulose, which favors the formation of dense mycelia that bind particle residues into cohesive networks [13]. Fungal species such as *Pleurotus* spp., *Ganoderma* spp., and *Trametes* spp. are commonly used due to their ability to bind various lignocellulosic residues, forming solid MBC materials with outstanding characteristics such as fire resistance, thermal stability, low thermal conductivity, and acoustic absorption capacity [13,14,15,16,17]. Multiple results reported in the review literature have demonstrated the versatility of the thermal and mechanical properties of MBCs, as well as the dependence of strength on fungal species and substrate type. Agro-industrial residues, such as sawdust, wood, and rye berries, that exhibit a high affinity for colonization by *Pleurotus* spp. and *Ganoderma* spp. demonstrate low thermal conductivity, making them effective in the production of thermal insulator MBCs [18,19,20,21]. In contrast, MBC production from *Trametes* spp. utilizing fiber materials such as hemp, flax waste, and wood residues supports the enhancement of mechanical properties [18,22,23]. Despite the various results reported above, the mechanical properties, dimensional stability, and water adsorption are considered factors that affect the structural integrity and durability of MBCs during an extended period, limiting the standardization of processes for large-scale building applications [24]. To overcome these constraints, natural alternatives such as fiber-reinforced materials, including jute, hemp, bamboo, and flax, in MBCs have been shown to significantly improve the mechanical properties of MBCs [18,25]. Additionally, pineapple fiber residues reduce water adsorption and enhance the dimensional stability of lignocellulosic substrates made from rice bran and coffee grounds [26]. In this context, the limited knowledge about the impact of lignocellulosic waste on the formation and physical, mechanical, and thermal characteristics of MBCs emphasizes the need to explore alternatives of new lignocellulosic substrates to optimize MBC properties, which can lead to building applications that align with a region’s waste management demands.

In the present research, we focus on developing MBC materials for constructive applications by combining two regionally available lignocellulosic residues: grass clippings (Kikuyu grass) from urban pruning activities and the pith of *Montanoa quadrangularis* (commonly known as Arboloco), a plant endemic to the Colombian–Venezuelan Andes. We use the fungal species *Ganoderma lucidum* as a binding agent to form the bio-composite’s mycelium network structure. Unlike conventional substrates, these agro-industrial residues do not compete with the agri-food or forestry sectors. Arboloco holds significant cultural and ecological value in Andean communities, where it has historically been used for firewood and vernacular construction during the Antioquian colonization [27], contributing to the insulating characteristics of the MBC. At the same time, Kikuyu grass serves as a fiber-reinforced material, which, according to the literature mentioned above, enhances the mechanical properties of the MBC. The experimental design assessed the effects of two particle size ranges of Arboloco pith (1.0–2.36 mm and 2.37–5.66 mm) and three variations of substrate formulation (Arboloco/Kikuyu grass) on the properties of mycelium-based composition. The physical properties (apparent density, shrinkage, and water absorption), mechanical properties (compressive strength, modulus of rupture, and elasticity), and thermal properties (diffusivity, conductivity, and specific heat capacity) of the mycelium-based composite, as well as its morphological and chemical properties (as observed using SEM and FTIR), were evaluated. This study contributes to a broader understanding of the potential applications of local lignocellulosic residues in non-structural building systems, providing a sustainable alternative to improve regional waste management challenges.

## 2. Materials and Methods

### 2.1. Organism and Spawn Preparation

**Figure 1:**
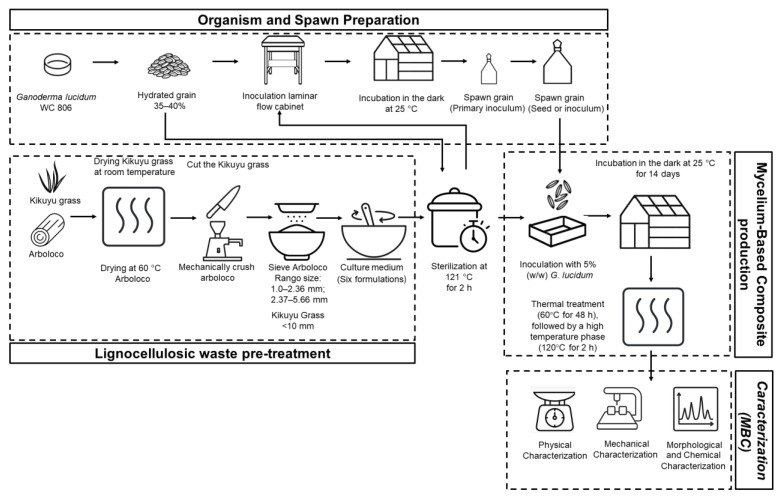
Schematic of the production and characterization process of a mycelium-based material (MBC) from lignocellulosic waste and *Ganoderma lucidum*. The diagram illustrates four main stages of the process: (1) Organism and spawn preparation involves cultivating Ganoderma lucidum WC 806 on sterilized grain until complete colonization. This process involves two phases: the preparation of a primary inoculum, followed by the production of fungal spawn intended for substrate inoculation. (2) Lignocellulosic waste pre-treatment occurs in parallel with the first stage. Both wastes are crushed, and after that, Arboloco is sieved into two particle ranges. Finally, the lignocellulosic waste is mixed based on the six formulations proposed in this work. (3) Mycelium-based composite production uses mixtures of Kikuyu grass (*Pennisetum clandestinum*) and Arboloco (*Montanoa quadrangularis*). This stage includes the preliminary conditioning of materials, substrate formulation, sterilization, and inoculation, followed by incubation until complete colonization of the material. (4) Material characterization involves thermal treatment for drying and inactivation, followed by physical, mechanical, and chemical analyses to evaluate the potential of the composite as an alternative biomaterial.

Figure 1 illustrates the schematic process for preparing the organism and spawn. A strain of *Ganoderma lucidum* WC806 obtained from the Pennsylvania State University Mushroom Culture Collection, USA, was used. It is available at the Technological Development Center of the Bioprocess and Agroindustry Plant of the University of Caldas and maintained on potato dextrose agar (PDA) at 4 °C with periodic transfers according to the protocols described by Montoya et al. [28]. The spawn for inoculating the solid substrate was prepared on wheat and hydrated with hot water up to 35–40% humidity. The hydrated wheat was packed in 200 g units in bioriented polypropylene bags, provided with a cotton filter on the bag top for gas exchange. The grain was sterilized at 121 °C for 2 h and inoculated with four 1 cm side pieces of mycelium extended on agar in a laminar flow cabinet. Inoculated bags were incubated in penumbra at 25 °C until complete colonization. The second stage continued with preparing the seed or inoculum packed with 1000 g of hydrated grain and inoculated with 4% (*w*/*w* fresh).

### 2.2. Culture Medium to Produce Mycelium-Based Composite (MBC)

To prepare culture media (Figure 1), lignocellulosic materials were selected: pith from the Arboloco tree (*Montanoa quadrangularis* Schultz Bip.) and Kikuyu grass (*Cenchrus clandestinus*). Both materials were collected from various locations in Manizales, Colombia (2280 masl, average temperature 17 °C, and 70% relative humidity). Arboloco (abbreviated A) and Kikuyu grass (abbreviated G) were dried at 60 °C and under ambient conditions, respectively. The dried Arboloco was mechanically crushed and sieved to separate particles into two size ranges: 1.0–2.36 mm (AL: Arboloco low particle size) and 2.37–5.66 mm (AH: Arboloco high particle size). Six culture medium formulations (Table 1) with an average moisture content of 94% were prepared to produce *Ganoderma lucidum* mycelium blocks. Each formulation was packaged in 2000 g bioriented polypropylene bags and sterilized at 121 °C for 2 h using the Tyndall method.

After sterilization, the substrate was transferred to acrylic molds disinfected with a 7% (*v*/*v*) formaldehyde solution. Four mold sizes were employed: M1 (6 cm × 6 cm × 4 cm; 15-cell mold), M2 (31 cm × 8.5 cm × 4 cm; 3-cell mold), M3 (12 cm × 6 cm × 7 cm; 6-cell mold), and M4 (33 cm × 33 cm × 4 cm). Twelve replicates were prepared for each combination of formulation and particle size. Each mold cell was inoculated with 5% (*w*/*w*) *G. lucidum* mycelium (wet basis relative to substrate mass) and introduced into a polypropylene bag with a cotton plug to enable gas exchange. The molds were incubated in the dark at 25 °C for 14 days to achieve complete mycelial colonization. At the midpoint of the incubation period, molds were inverted to ensure homogeneous mycelial coverage. A stepped thermal treatment was applied to inactivate the *G. lucidum* mycelium: an initial low-temperature phase (60 °C for 48 h) followed by a high-temperature phase (120 °C for 2 h).

### 2.3. MBC Physical Characterization

#### 2.3.1. Apparent Density

The MBC samples were previously dried at 50 °C for 96 h. The apparent density was calculated by weighing the dried samples and calculating their volume according to the ASTM D3573-20 standard protocol [29]. The formula for dry density was ρ = m/V, where ρ is the dry density in kg/m^3^, m is the mass of the composites in kg, and v denotes the sample volume in m^3^. At least four replicates were recorded for each experimental condition.

#### 2.3.2. Water Absorption

The water absorption test was developed based on the standard method ASTM D570-22 [30]. Before the composite was dried at 50 °C for 24 h, the initial composite mass was determined after cooling. Subsequently, the samples were submerged in deionized water for 48 h and weighed. Due to the low density of the MBC material, it was forced to remain submerged using wire mesh and crystal balls. The weight change was calculated using the following equation:

Mass Change (%) = (W − D)/D × 100,(1)

where W = wet mass (kg) and D = dry mass. Each experimental condition was carried out five times.

#### 2.3.3. Shrinkage

The shrinkage of the sample was determined and calculated based on the wet and dry volumes using the following expression:

Shrinkage percentage (%) = [(V_1_ − V_2_)/V_1_] × 100, (2)

where V_1_ = the wet volume of the sample and V_2_ = the dry volume of the sample. Thirty-six replications evaluated each experimental condition.

### 2.4. MBC Morphological and Chemical Characterization

The functional groups for each MBC were assessed by Fourier Transform Infrared Spectroscopy (FTIR) using the Thermo Scientific model Nicolet iS5 (Thermo Fisher Scientific, Waltham, MA, USA), covering a range of 400–4000 cm^−1^. The microscopic morphology of the interior of the composite (approximately 3 mm from the surface) was photographed and examined with a scanning electron microscope (SEM), TENSCAN VEGA 4th generation, (TESCAN, Brno, Czech Republic) using an accelerating voltage of 15 kV.

### 2.5. MBC Mechanical Characterization

#### 2.5.1. Compression Strength

The compressive strength tests were determined according to procedure B of the standard norma ASTM C165-23 [31] utilizing a HUMBOLT HM-5030 F instrument (Humboldt Mfg, Elgin, IL, USA) with a 5 kN load capacity. Each sample (5 × 5 × 2.2 cm) was compressed until it achieved 50% of its original thickness at 12.5 mm/min. The contact surface on the plates was not perfect due to the rough surface of the samples. The loads and tenses were recorded periodically. An initial pre-load of 2% of the final load was considered to ensure the homogeneity of the samples.

#### 2.5.2. Flexural Strength

The flexural strength was measured based on procedure B of the standard norma AST C203-22 [32]. A rectangular cross-section (27 × 6 × 2.9) was exposed to apply force through the 3-point bend test at 36.55 mm/min speed using a SHIMADZU AG-X instrument (Shimadzu Corporation, Kyoto, Japan) with a load cell of 10 kN. The force was applied to the sample until it reached fracture or a maximum deflection of 22 mm. The Modulus of Rupture (MOR) and Modulus of Elasticity (MOE) were calculated using the following equation.

Maximum deflection was calculated by:

D = (εL^2^)/6d, (3)

where

D: deflection at the center of the sample (mm);
  ε: strain (mm/mm);
  L: support span (mm);
  d: depth of the beam (mm).

The modulus of rupture (MOR) and modulus of elasticity (MOE) were calculated using the following equations:

MOR = 3PL/(2bd^2^), (4)

where

P: load at a given point on the load–deflection curve (N);
  b: width of the beam tested (mm);

and

MOE = (PL^3^)/48ID, (5)

where I: moment of inertia (mm^4^).

### 2.6. MBC Thermal Characterization

The thermal properties, conductivity, diffusivity, and specific heat were recorded using the ISOMET 2114 instrument (APPLIED PRECISION Measurement and test solution, Bratislava, Slovakia) following the standard ASTM-D5334 [33]. The cylindrical samples (10 × 4.5 × 4.5 cm) were dried at 50 °C until they reached a constant weight. Subsequently, the samples were wrapped with an ultra-thin sheet of low-density polyethylene to preserve their anhydrous state. They were then kept at a constant temperature of 22 °C for 4 h before the thermal measurement. The needle probe was inserted into each prismatic sample to a minimum depth of 8 cm, ensuring proper contact. Finally, the thermal data were recorded.

## 3. Results and Discussion

### 3.1. Morphological Characteristics

**Figure 2:**
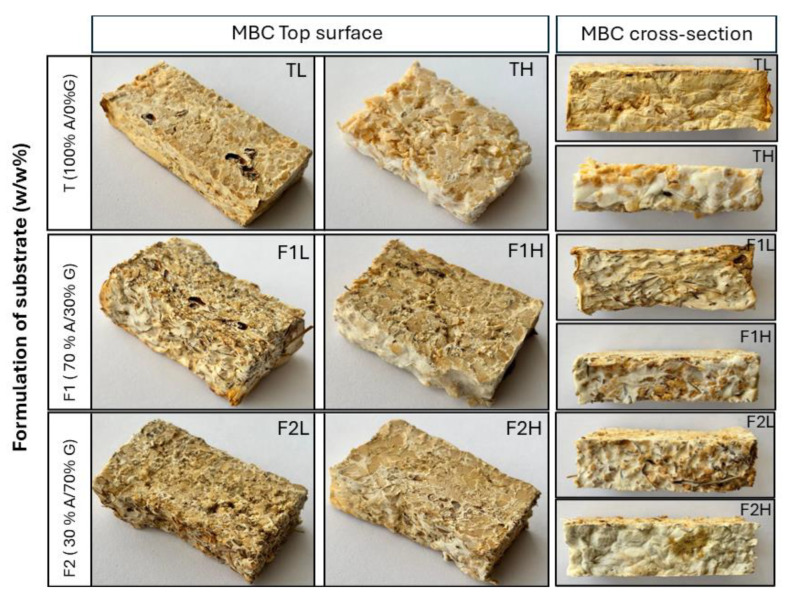
Photographs of the mycelium-based composite at the top surface and cross-section under six different formulations—TH, TL, F1L, F1H, F2L, and F2H—where A: Arboloco pith; G: Kikuyu grass; F1: 70% A/30% G; F2: 30% A/70% G; and Arboloco pith particle sizes are L: 1.0–2.36 mm and H: 2.37–5.66 mm.

**Figure 3:**
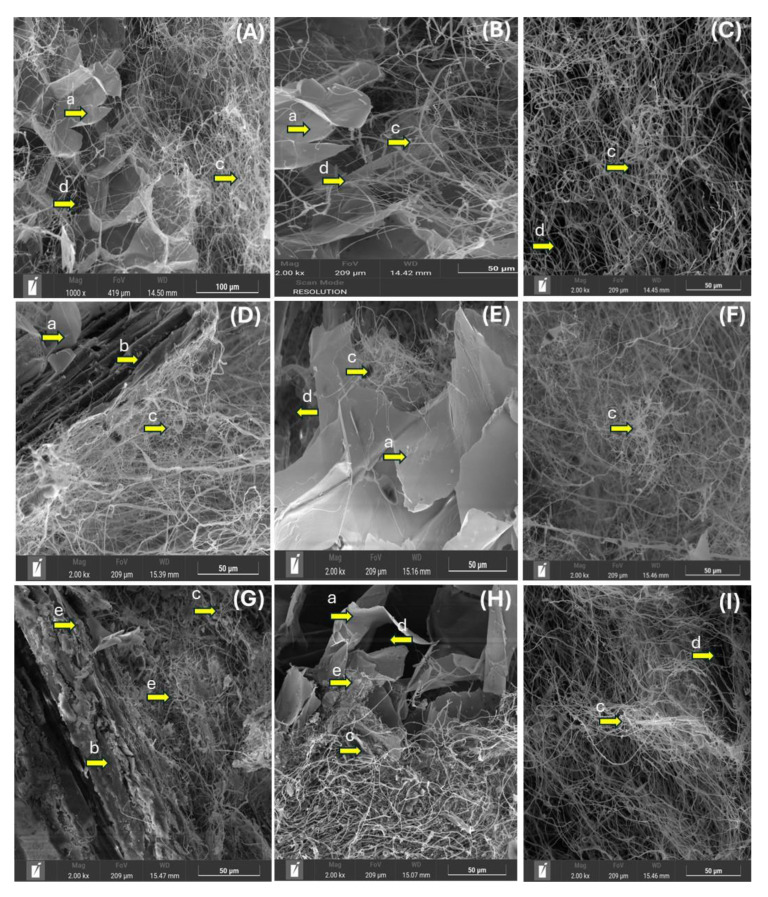
SEM images of the mycelium-based composite (MBC) considering the following formulations: (**A**–**C**) 100% A/0 G; (**D**–**F**) 70% A/30% G; (**G**–I) 30% A/70% G. Characteristics of the MBC are based on the following aspects: (a) Arboloco pith, (b) Kikuyu grass, (c) hyphae structure, (d) air void, and (e) exopolysaccharides (EPSs).

Figure 2 and Figure 3 illustrate the morphological characteristics of mycelium growth. The analysis of the top surface reveals a dense mycelial coverage with pronounced surface irregularities, particularly in conditions containing some Kikuyu grass (Figure 2). Cross-sectional images show that high Arboloco particle sizes under conditions TH and F1H favor dense internal hyphal colonization, occupying mainly the inner air voids of the biomaterial, while conditions of low particle size (TL and F1L) evidence a complete hyphal coverage (both inner and outer). The F2 formulation shows an inverse trend, where a tightly packed structure associated with a higher mycelium yield was associated with larger particle sizes (F2H).

The analysis of SEM images (Figure 3A–I) demonstrates noticeable elongated, highly branched, and ramified hyphae with randomly arranged and oriented growth. The T1 formulation (100% A/0% G) exhibits a low-density mycelium surface layer around the substrate, with no hyphal adherence (Figure 3A–C). At the same time, a higher number of air voids is observed in the material. Nevertheless, increasing the proportion of Kikuyu grass favors the densification of the mycelium. In the case of F1, hyphae growing around the substrate without adhering to the material (Arboloco pith and Kikuyu grass) were detected (Figure 3D–F). In contrast, Figure 3G–I show a higher densification of hyphae and firm adherence to the substrate, developing a hyphal bundle embedded in a polysaccharide matrix (Figure 3G,H) as observed by Pesciaroli et al. [34] These characteristics seem to be correlated with the complete and tight mycelium structure observed with the F2 formulation (Figure 2).

**Figure 4:**
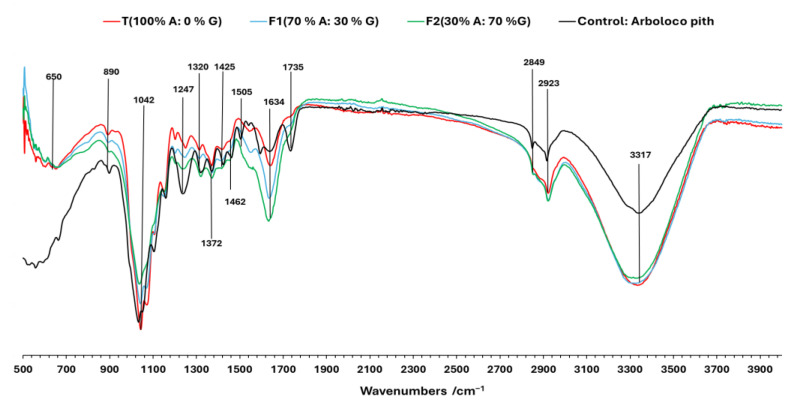
FTIR spectra of MBC under different formulations: 100% A/0%G, 70% A/30% G, 30% A/70% G, and control composed of uncrushed Arboloco pith without colonization. A: Arboloco pith; G: Kikuyu grass. Previously, the transmittance signals were standardized.

Fourier transform infrared spectroscopy (FTIR) analysis of the MBC colonized by *G. lucidum* (Figure 4) revealed distinct structural modifications compared to non-colonized raw material (uncrushed Arboloco pith) obtained in previous studies in our laboratory [35]. The peaks in the fingerprint and their functional group interactions are assigned based on previous studies FTIR studies on the changes in wood chemistry following decay by brown-rot and white-rot fungi [18,36]. A progressive decrease (T1 < F1 < F2) in intensity at 1042 cm^−1^ (C-O-C and C-O stretching vibrations in cellulose and hemicellulose) and 890 cm^−1^ (C–H deformation of cellulose) demonstrates fungal hydrolysis of polysaccharides in cellulose, while the decline of the 1735 cm^−1^ band (ester C=O stretching) indicates hemicellulose deacetylation [18,37]. A notable change was observed at 1247 cm^−1^, where the strong peak in non-colonized Arboloco material declines after fungal colonization, indicating the degradation of aryl-ether and ester linkages in lignin and hemicellulose, respectively. In concordance with the enzymatic white-rot fungi, the complete disappearance of peaks at 1425 cm^−1^ (CH_2_ bending in crystalline cellulose), 1596 cm^−1^, and 1505 cm^−1^ (aromatic C=C vibrations in lignin) in the MBC samples provided further evidence of the structural breakdown of lignin and complex carbohydrate bonds. On the other hand, the progressive broadening and elongation of the 1634 cm^−1^ peak (C=O stretching) shows the accumulation of oxidized lignin derivatives (carbonyl and quinoid structure) associated to components such as chitin and glucans, while the broadening and intensification of the 3317 cm^−1^ (O-H/N-H stretching) band with respect to the control (uncrushed Arboloco pith) suggests increased hydrophilicity resulting from both the exposure of cellulose hydroxyl groups after lignin removal and the water-binding capacity of fungal mycelium. The inverse relationship between peaks at 1042 cm^−1^ and 1634 cm^−1^ (Figure 4) suggests the accumulation of exopolysaccharides, as observed in Figure 3G–I under the F2 formulation. Previous studies support this behavior, in which a decline in the peak at 1042 cm^−1^ is attributed to the depolymerization of cellulose into monomeric sugars, which fungi metabolize to synthesize the EPS as β-glucans [18], while an increase in the peak at 1634 cm^−1^ is linked to oxidized lignin derivatives and the simultaneous production of carbonyls and glucuronic acid (a component of fungal β-glucans) [38,39]. These spectroscopic changes collectively illustrate the unique ability of white-rot fungi to degrade all major lignocellulose components through a combination of hydrolytic and oxidative mechanisms while simultaneously introducing fungal exopolysaccharides into the substrate matrix.

### 3.2. Physical Properties

**Figure 5:**
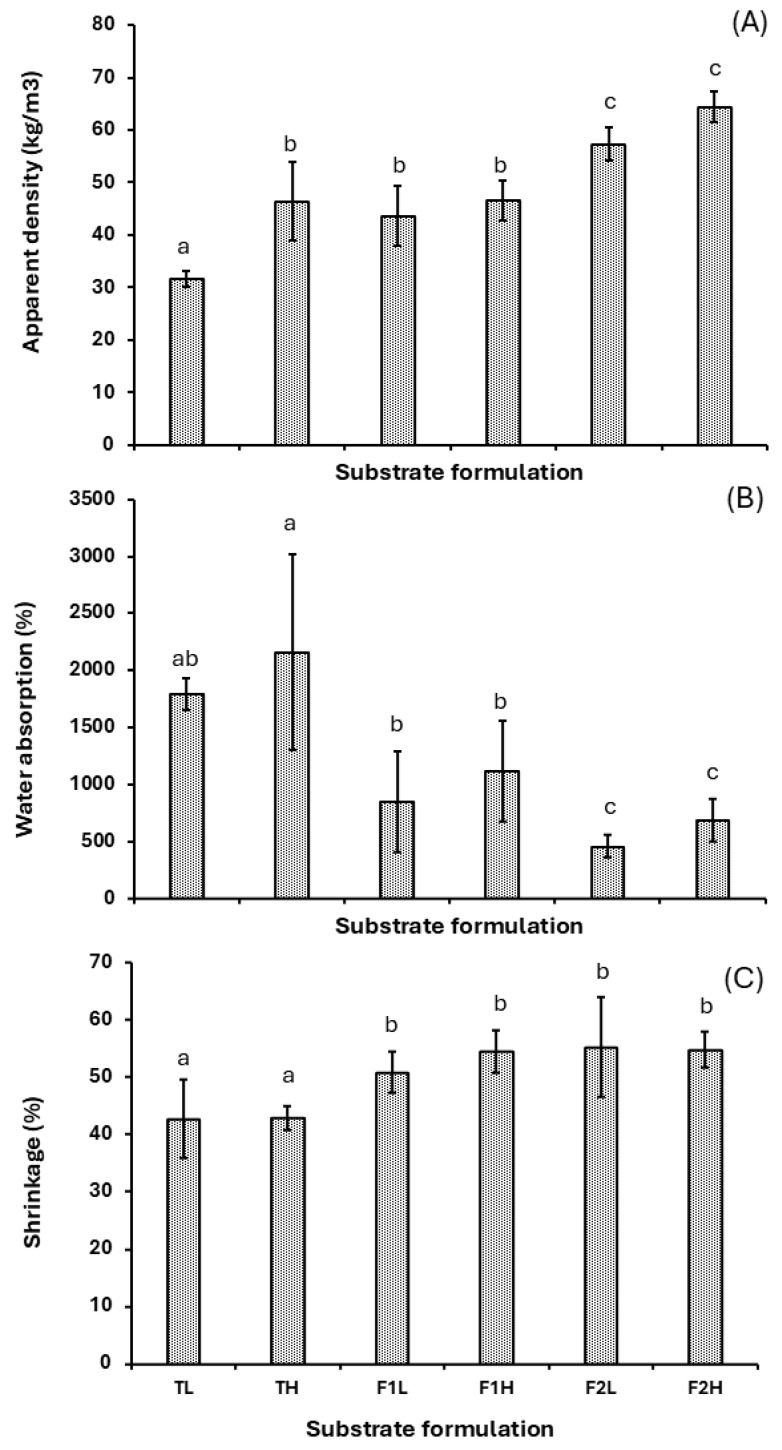
Physicochemical properties of MBC: (**A**) apparent density, (**B**) water absorption, and (**C**) shrinkage under different substrates. A (Arboloco pith), G (Kikuyu grass), and particle sizes L (1.0–2.36 mm) and H (2.37–5.66 mm). Substrate formulations T (100% A/0% G), F1 (70% A/30% G), and F2 (30% A/70% G). The results are shown as the mean ± SD. The lowercase letters above bars indicate significant differences between substrate formulations (Tukey, *p* ≤ 0.05, *n* ≥ 10).

The analysis of the physical properties of the MBC considered three parameters—apparent density, shrinkage, and water adsorption—which were recorded throughout the experiment. Figure 5A shows that the biomaterial composed of 100% Arboloco achieved the lowest average density of 31 ± 1.6 kg m^−3^. These trends are reversed as the percentage of Arboloco decreases from 100 to 30%, while the Kikuyu grass content increases from 0 to 70%, causing a rise in the average density up to 60.4 ± 4.5 kg m^−3^. Comparatively, the MBC density values were 3.3-fold lower than those reported for lignocellulosic substrates such as beech sawdust [12], wheat bran [40], and rice and wheat straw [41] used in *G. lucidum* mycelium composite (260 to 370 kg m^−3^). The low apparent density of the Arboloco–Kikuyu grass MBC (31–60.4 kg m^−3^) positions it as a lightweight biomaterial, comparable to natural insulators like sheep’s wool (30–114 kg m^−3^) [42] and synthetic alternatives such as glass wool (10–100 kg m^−3^) [43]. This lightweight property suggests its suitability for applications in non-structural and building envelopes, being typically used for thermal insulation materials. The inverse correlation between the Arboloco pith content and apparent density is probably linked to low-density mycelium growth and the high presence of air voids (Figure 3), causing an increase in the MBC porosity, a typical characteristic of lightweight biomaterials. A similar trend was observed in the water absorption (Figure 5B), achieving values above 1700% after 48 h of water immersion under the formulations TL and TH, surpassing raw uncrushed Arboloco pith without mycelium colonization (425%) previously evaluated in our laboratory [35]. This trend could be promoted by the increase in the porosity of the bio-composite, a consequence of the Arboloco particle size distribution and the hygroscopic properties of the mycelium, which accelerate the hydraulic penetration and lower drag force, increasing the water intake of the MBC [44]. However, increasing the fiber content in the substrate triggers a sharp decline in the average water absorption, reaching 556 ± 184% in the F2 conditions and achieving consistent values as reported in the MBC literature, which ranges from 40 to 580 [45]. Regarding shrinkage post-drying, the presence of fiber in the substrate (F1–F2), independent of particle size, leads to a maximum shrinkage percentage of 53.6 ± 5.6%, exceeding that of substrates with only Arboloco (T1–T2) by 10%. This shrinkage percentage was markedly higher than other studies of MBCs utilizing sawdust (6.8–14.5%) [46], hemp and flax (7–15%) [26] as a mycelium growth support. According to Islam et al. [47], the random direction of hyphal growth in the mycelium microstructures, as observed in our MBC (Figure 3), is evidence of intense heterogeneous deformation, resulting in an increase in the shrinkage properties. These high shrinking and water absorption properties make it difficult to predict the geometric and dimensional properties after drying treatment, becoming a challenge in this novel biomaterial. Hot pressing post-treatment on the *Trametes versicolor* MBC has demonstrated a decrease in the hydrophobicity of the mycelium and porosity of the bio-composite [22], which favors the diminishment of water uptake and shrinking properties. This alternative arises as an option to implement in our material that can contribute to overcoming the constraint associated with forming regular constructive structures for implementation in massive-scale production.

### 3.3. Mechanical Properties

**Figure 6:**
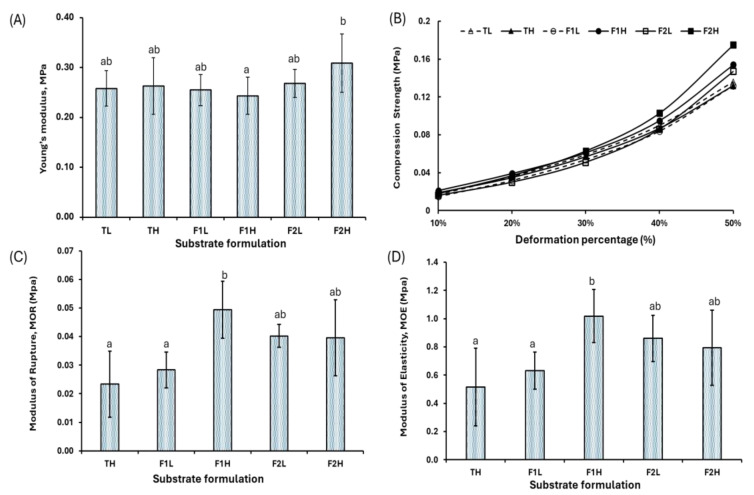
Mechanical properties of MBC: (**A**) Young’s modulus, (**B**) compressive strength, (**C**) modulus of rupture (MOR), and (**D**) modulus of elasticity (MOE) under different substrates A (Arboloco pith) and G (Kikuyu grass) and particle sizes L (1.0–2.36 mm) and H (2.37–5.66 mm). Substrate formulations T (100% A/0% G), F1 (70% A/30% G), and F2 (30% A/70% G). The results are shown as the mean ± SD. The lowercase letters above bars indicate significant differences between substrate formulations (Tukey, *p* ≤ 0.05, *n* ≥ 6).

The mechanical properties of the developed MBC showed no significant variations in the Young’s modulus of compression due to the composition of the raw materials used (Figure 6A). The average value obtained was 0.264 ± 0.045 MPa, indicating low stiffness but notable elastic capacity. This behavior suggests that the material can absorb energy and deform without compromising its structural integrity. Such behavior aligns with prior findings highlighting the potential of lightweight biomaterials with low stiffness but high energy absorption capacity, such as cork [48]. The compressive strength analysis showed a progressive increase with the deformation percentage, with average values ranging from 0.017 MPa at 10% to 0.146 MPa at 50% deformation (Figure 6B). This trend reflects a nonlinear strengthening response under higher compressive loads. Regarding compressive strength, the formulations exhibited consistent performance, with formulation F1H achieving strengths of 0.0031, 0.0589, 0.0934, 0.1465, and 0.154 MPa at deformation levels of 10%, 20%, 30%, 40%, and 50%, respectively. On the other hand, increasing the Kikuyu grass content (formulation F2) improved the material’s performance at higher deformation levels, reaching an average compressive strength of 0.1686 MPa at 50% deformation. Although these values are consistent with those reported for low-density mycelium-based materials, such as the 0.024 MPa reported by Vidholdová et al. [49] at 20% deformation using *Trametes versicolor* and spruce wood, they are notably lower than those observed in densified composites. For instance, Sisti et al. [50] achieved compressive strengths ranging from 0.04 to 0.08 MPa at 25% deformation to 0.53 to 1.00 MPa at 75% deformation using wheat bran and industrial fibers inoculated with *Basidiomycetes*, while Etinosa et al. [51] reported strengths of 0.9 MPa at 10% deformation using *Pleurotus ostreatus* and a substrate enriched with calcium and starch. In contrast, recent research suggests that the relationship between density and compressive strength in mycelium-based materials is not always direct. Peng et al. [52] observed a positive correlation between these properties. In contrast, the results of our study did not show significant differences in the compressive strength despite an increase in the apparent density due to substrate composition. This indicates that the mechanical properties remain unchanged under low-density conditions (<0.1 g/cm^3^). This aligns with the results of Vidholdová et al. [49], who attributed the low compressive strength of porous mycelium-based materials to increased air voids, which contribute to early-stage deformation. Compared to other studies, composites with higher densities and base materials such as beech sawdust or natural fibers have demonstrated significantly higher strengths. For example, Sivaprasad et al. [53] reported compressive strengths of up to 2.49 MPa at 20% deformation using *Pleurotus ostreatus* and *Ganoderma lucidum* grown on beech sawdust and other agro-industrial substrates. At higher deformations (>50%), these strengths can be up to five times higher than those observed in this study, highlighting the critical role of substrate selection and densification processes in mechanical performance. Regarding flexural properties (Figure 6C,D), the MBCs exhibited a modulus of rupture (MOR) of 0.0339 ± 0.0145 MPa and a modulus of elasticity (MOE) of 0.75 ± 0.3237 MPa. Although these values fall within the range reported for non-pressed mycelium-based composites with a MOR from 0.05 to 0.29 MPa [54], these are relatively low compared to other studies. For instance, Rigobello & Ayres [55] achieved a MOE of 3.32 MPa and a MOR of 0.306 MPa using *Ganoderma lucidum* and European beech particles. Additionally, some formulations (F1 and F2) outperformed samples TH, suggesting that the substrate composition can influence flexural properties. However, variations between different particle sizes were not significant. The combination of low stiffness and high resilience observed in this study highlights the potential of mycelium-based composites for applications requiring flexibility and energy absorption, such as impact-resistant panels and safety components in the automotive industry. While the mechanical performance observed is relatively low compared to other MBCs reported in the literature, the biodegradable and lightweight nature of these materials positions them as sustainable alternatives for various industrial applications. Additionally, the limited dependence of mechanical properties on material density under low-density configurations could be advantageous for developing lightweight materials for non-structural applications.

### 3.4. Thermal Properties

**Figure 7:**
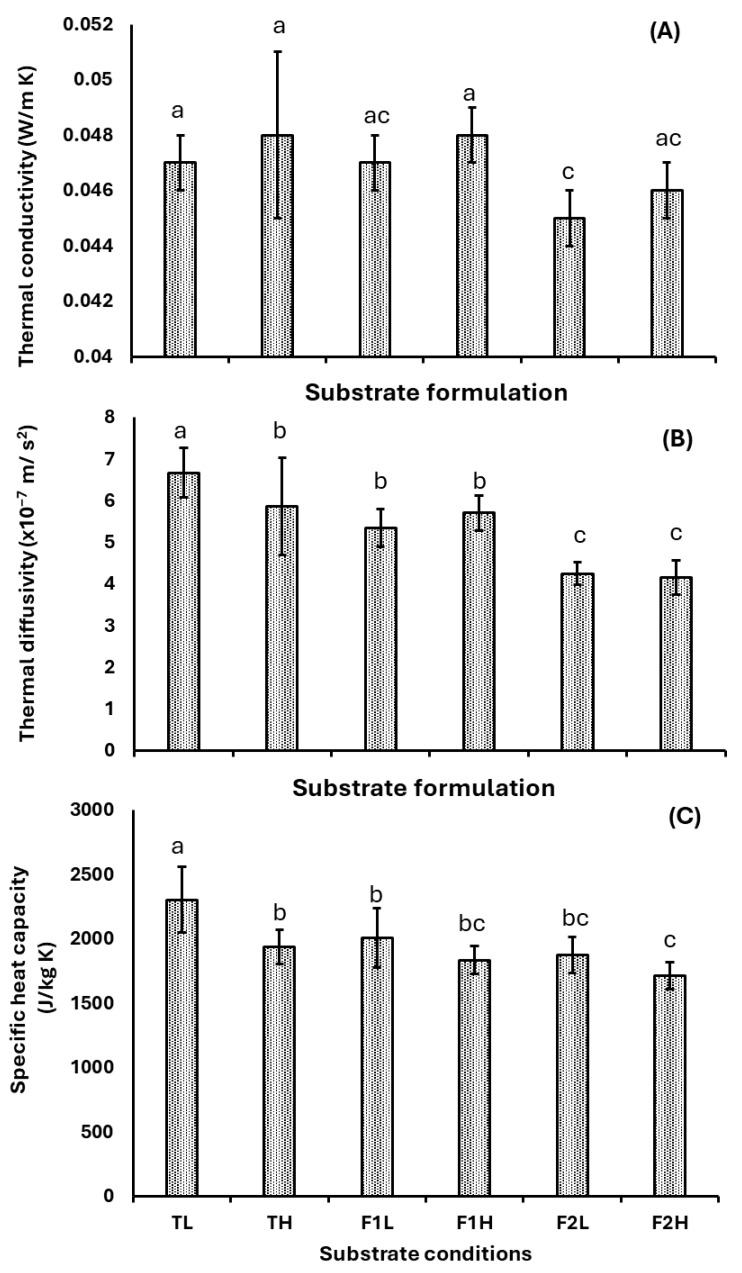
Thermal properties of the MBC: (**A**) thermal conductivity, (**B**) thermal diffusivity, and (**C**) specific heat capacity under different substrates T (Arboloco) and G (Kikuyu grass) and particle sizes L (1.0–2.36 mm) and H (2.37–5.66 mm). Substrate formulations T (100% A/0% G), F1 (70% A/ 30% G), and F2 (30% A/70% G). The results are shown as the mean ± SD. The lowercase letters above bars indicate significant differences between substrate formulations (Tukey, *p* ≤ 0.05, *n* ≥ 9).

Previous studies in our laboratory on the raw substrate of uncrushed Arboloco pith have demonstrated its promising use as a building insulation material due to its low thermal conductivity of 0.044 W m^−2^ K [35]. Based on the results observed in Figure 7A, this thermal conductivity was conserved in the MBC, reaching an average value of 0.047 ± 0.002 W m^−1^ K^−1^ throughout the experiment. A comparative analysis of the experimental conditions shows that the particle size of the crushed Arboloco pith did not significantly affect the thermal conductivity across formulations (T, F1, and F2). However, conditions with a high proportion of Kikuyu grass F2 (30% A/70% G) exhibit the lowest thermal conductivity at 0.045 W m^−1^ K^−1^. These outstanding thermal performances are typically attributed to materials with a low density and high porosity, as observed in our experiments (<80 kg m^−3^), being comparable with the properties of commercial lightweight insulation boards (density below 150 kg m^−3^) such as expanded polystyrene and cork, which exhibit thermal conductivity values of 0.03–0.05 W m^−1^ K^−1^ [7,56]. For MBCs, prior studies emphasize that thermal properties are strongly influenced by the substrate composition more than the fungal species. For instance, lignocellulosic substrates such as birch wood [23], bamboo fiber [57], sawdust [58], and coffee silver skin [59] bonded by the mycelium of *Ganoderma lucidum*, *Pleurotus ostreatus*, and *Trametes versicolor* have reported wide thermal conductivity ranges from 0.04 to 0.18 W m^−1^ K^−1^. In this sense, our MCB demonstrates competitive thermal insulation properties, positioning it as a sustainable, low-cost biomaterial for construction applications.

Thermal diffusivity and specific heat capacity show a decline in the average values from 6.6 ± 0.6 × 10^−7^ to 4.1 ± 0.4 ^3^ 10^−7^ m^2^ s^−1^ and 2303 ± 254 to 1714 ± 105 J/kg K, respectively, as the proportion of Kikuyu grass increases in the substrate (Figure 7B,C). Prior studies on plaster and high-density polyethylene (HDPE) material demonstrated similar trends, where the addition of wood, wheat, and flax fiber reduced the thermal diffusivity of the bio-composite [60,61]. Furthermore, the highest thermal diffusivity patterns under TL and TH conditions suggest a direct association with the low mycelium densification (Figure 3A–C) and apparent density properties (Figure 5), leading to highly porous structures and air-entrapped voids. These microstructural features enhance heat retention while lowering the thermal diffusivity. The specific heat capacity (Figure 7C) shows a higher performance than commercial insulating materials such as expanded polystyrene, polyurethane foam, and wood fiber (800–1500 J/kg K) [62,63], even reaching competitive properties as observed with natural composites of bamboo fibers, cotton waste, or epoxy–natural fiber (1300–1800 J/kg K) [64,65]. These characteristics suggest that our MBC performs better in absorbing and storing heat, reducing temperature fluctuations, and enhancing indoor thermal stability and comfort, which is a critical advantage for green building design. In contrast, mycelium-based composites (MBCs) fabricated from *Ganoderma resinaceum* [66], *Pleurotus ostreatus* [19], and *Ganoderma Lucidum* (previous studies in our laboratory), using rye berries and wheat straw as substrates, exhibited a specific heat capacity ranging from 4100 to 6500 J kg^−1^ K^−1^, reaching values threefold higher than those observed across our study. These findings suggest that thermal properties are primarily determined by substrate composition rather than fungal species. Future studies should prioritize optimizing substrate formulations (e.g., blends of lignocellulosic agro-industrial wastes) to improve thermal performance while aligning with circular economy principles and carbon neutrality objectives.

## 4. Conclusions

- The development of mycelium-based composite materials (MBCs) using *Ganoderma lucidum* and Colombian agro-industrial wastes (Arboloco and Kikuyu grass) illustrates significant potential as a lightweight thermal insulation material with a low environmental impact, consistent with the principles of sustainable construction.
- Formulation F2, consisting of 30% Arboloco and 70% Kikuyu grass, exhibited an outstanding thermal performance, with a thermal conductivity of 0.045 W m^−1^ K^−1^ and a specific heat capacity of 1714 ± 105 J kg^−1^ K^−1^. Moreover, the low apparent density (60.4 ± 4.5 kg·m^−3^) positions it as a promising lightweight insulating material that could serve as an alternative to synthetic insulation materials like expanded polystyrene. These results establish our MBC as a viable energy-efficient option.
- Despite the thermal and ecological advantages of these biomaterials, significant challenges, including high volumetric shrinkage (53.6%) and elevated water absorption (556 ± 184%), must be addressed, as they limit the materials’ direct applicability without additional treatment. These findings highlight the necessity of incorporating post-processing techniques, such as hot pressing, to enhance dimensional stability and developing strategies aimed at reducing water absorption capacity.
- Future research should focus on substrate optimization by exploring hybrid combinations of lignocellulosic biomass to enhance mechanical performance without compromising eco-design principles. Additionally, the standardization of growth and treatment protocols will be essential for achieving controlled and reproducible scaling of MBC production.

## Figures and Tables

**Table 1:** Substrate formulation conditions based on the *w*/*w* % of the substrate on a dry basis and Arboloco particle size distribution.

Particle Size, mm	F1 (70%A/30% G)	F2 (30% A/70% G)	T (100% A/0% G)
L: 1.0–2.36	70	30	100
H: 2.37–5.66	30	70	0

Six formulations: TH, TL, F1L, F1H, F2L, F2H, considering A: Arboloco, G: Kikuyo grass, L: low particle sizes, H: high particle sizes.

## Data Availability

The original contributions presented in the study are included in the article, further inquiries can be directed to the corresponding author.
